# Caring for the frail: a qualitative study in an orthopaedic setting

**DOI:** 10.1186/s12877-026-07479-7

**Published:** 2026-04-16

**Authors:** Amanda Hammer, Ylva Nilsagård, Erika Fjordkvist, Maria Hälleberg Nyman

**Affiliations:** 1 Department of Geriatrics, Faculty of Medicine and Health, Örebro University, Örebro, SE-701 82 Sweden; 2 University Health Care Research Centre, Faculty of Medicine and Health, Örebro University, Örebro, SE-701 82 Sweden; 3 School of Health Sciences, Department of Orthopaedics and Hand surgery, Faculty of Medicine and Health, Örebro University, Örebro, SE-701 82 Sweden; 4 School of Health Sciences, Örebro University, Örebro, SE-701 82 Sweden

**Keywords:** Clinical Frailty Scale (CFS), Frailty, Healthcare professionals, Qualitative research, Thematic analysis

## Abstract

**Background:**

The number of frail individuals is increasing, and the proportion of frail patients in hospital settings is high. Assessing patient frailty as a part of hospital care can guide clinicians in determining which interventions are needed and what to prioritise. Orthopaedic in-patients often experience both pain and mobility limitations, which, in combination with frailty, make these patients particularly vulnerable. There is limited knowledge regarding how staff working in orthopaedic acute care units understand frailty and how they adjust their clinical practices in response. The aim is to understand how healthcare professionals in an orthopaedic acute care unit perceive frailty and possibly adapt their clinical practices in response to it.

**Methods:**

The study adopted a qualitative design and included 16 healthcare professionals from two hospitals. Semi-structured individual face-to-face interviews were conducted, digitally recorded, transcribed verbatim, and subsequently analysed using thematic analysis with an inductive approach.

**Results:**

Two themes were developed during the analysis mirroring the healthcare professionals’ perceptions. The first is “*Considering an individual frail”* supported by the subthemes ”*Describing* *frailty in different ways”*,* “Recognising vulnerability”*,* “Identifying the need for support”*, and *“Acknowledging frailty in different contexts****”***. The second is *“Caring for the frail”*, supported by the subthemes “*Understanding the concept of frailty”*,* “Assessing frailty”*,* “Taking frailty into account”*,* “Being one step ahead”*, and *“Having resource awareness”.*

**Conclusions:**

This study revealed that healthcare professionals perceive frailty as a broad and multidimensional concept that requires clearer specification to guide appropriate interventions. Participants emphasised that care is shaped primarily by individual needs rather than by frailty assessment, reflecting a person-centred approach. They also expressed a strong sense of responsibility toward frail patients and viewed hospital admission as an opportunity to identify and address unmet needs at home. While tools such as the Clinical Frailty Scale were viewed as limited in terms of capturing the full complexity of frailty, they were described as helpful for drawing attention to frailty and prompting deeper reflection.

**Trail registration:**

https://researchweb.org/is/fourol/project/281574 Reg. no 28 15 74.

**Supplementary Information:**

The online version contains supplementary material available at 10.1186/s12877-026-07479-7.

## Background

The prevalence of frailty is increasing across the population among different age cohorts [1], and it represents a major challenge for both healthcare delivery and societal structures [2]. The World Health Organization (WHO) defines frailty as *“a progressive age-related decline in physiological systems that results in decreased reserves of intrinsic capacity*,* which confers extreme vulnerability to stressors and increases the risk of a range of adverse health outcomes”* [3]. Frailty is highly prevalent in the older population [1] and older adults exhibit a pronounced risk of developing frailty over time [2]. Also, the prevalence of frail individuals in hospital settings is high [4]. Although frailty is predominantly studied in older adults [5], research shows that it can also be present in younger individuals [6].

Orthopaedic in-patients most often experience some degree of both mobility limitation and pain, which may distinguish them from patients in several other acute medical specialties [7]. The length of hospital stay for orthopaedic diagnoses varies [8]. Hospital stays have decreased in recent years, for one example the length of stay for patients undergoing hip fracture surgery in Sweden decreased from 8.4 days in 2017 to 6.2 days in 2024 [9]. The combination of patient frailty and the accelerated pace of acute care processes [10] requires healthcare professionals to quickly and fully understand the specific needs and vulnerabilities of this patient group [11]. Many orthopaedic injuries are associated with an expectation of full or partial recovery but regardless of the expected prognosis, the quality of care provided at the ward [12], and the handover to the next level of care are of great importance [13]. Healthcare professionals working with frail orthopaedic patients play a crucial role in ensuring optimal care for this vulnerable patient group [12]. Adopting a person-centred approach provides enhanced conditions for the patients [14, 15]. To provide person-centred care; staff need supportive conditions, including adequate staffing, reasonable workloads, and sufficient time to engage with patients [16]. Assessing frailty in patients has become more usual in different healthcare settings in recent decades. Frailty assessment can serve as one of several tools to guide the focus of a person-centred approach [15]. There are several methods and scales available for frailty assessment, and the Clinical Frailty Scale (CFS), remains one of the most widely adopted [17] and clinically validated measuring tools [18]. The CFS is a 9-point ordinal scale, with higher scores indicating increasing levels of frailty; it ranges from 1 (Very fit) to 9 (Terminally ill) [19]. The CFS is widely used by staff in acute care setting to assess patient frailty [20]. However, an earlier interview study with healthcare professionals from various hospital wards revealed that the perceived benefits of frailty assessment were limited and that communication about frailty posed challenges [21]. Further, it was shown that healthcare professionals have different understandings of the concept of frailty depending on profession, level of experience, and training [22].

This makes it relevant to examine whether healthcare professionals understanding of the concept influences their perceptions of frailty and how it may shape the ways in which they adapt their clinical practice. To the best of our knowledge, no previous studies have investigated how healthcare professionals perceive frailty and adjust their work accordingly in an orthopaedic context. The aim is therefore to understand how healthcare professionals in an orthopaedic acute care unit perceive frailty and possibly adapt their clinical practices in response to it. Understanding how healthcare professionals perceive frailty may provide insights that could inform person centred care and quality improvement efforts.

## Methods

The aim of this study was to describe healthcare professionals’ perceptions of the concept of frailty and how they adapt their work accordingly. The study used a qualitative design and thematic analysis with an inductive approach [23]. Data were collected using a study-specific semi-structured individual interviews (Additional file 1). The study was approved by the Swedish Ethical Review Authority (Dnr: 2023-07274-01).

### Study settings

This study was part of a larger project, conducted at two orthopaedic sites in one county in Sweden, in which the intervention consisted of a customised healthcare practice for older adults (≥ 65 years) admitted for osteoporosis fractures (hip fractures, pelvic fractures or vertebra compression fractures). According to the project routine, all patients were to be assessed for frailty using the CFS and receive care customised to their frailty level, including medical treatment, nursing and rehabilitation. In addition, all patients were ensured the same level of basic care, ensuring equal evidence-based care.

One ward has 21 inpatient beds, admits patients from 18 years and older and has a certain trauma specialisation which contributes to a diverse patient clientele and age. This ward has about 21 nurses, 31 assistant nurses, 3 physiotherapists, 3 occupational therapists and 1 counsellor. The occupational therapists and counsellors were employed at the ward and the physiotherapists at a separate department. The other ward has 16 inpatient beds, admits patients from 18 years and older and is the unit primarily responsible for the care of hip fracture patients, which results in more older adults. This ward has about 12 nurses, 17 assistant nurses, 1 physiotherapist, 1 occupational therapist and 0,25 counsellors. There were 70 physicians in the section, and they work across the units, only one physician is permanently stationed on the ward. The physiotherapists, occupational therapists and counsellors were employed in a separate department. Patients with hip fractures are cared for at both wards. According to registry data from Rikshöft for 2025, 95 patients underwent surgery for hip fracture at one unit, with a mean age of 81 years; 61% were women and the mean length of stay was 8 days [9]. At the other unit, 361 patients underwent surgery for hip fracture, with a mean age of 82 years; 62% were women and the mean length of stay was 7 days [9].In Sweden, healthcare is predominantly financed through taxation, with patient fees accounting for only a minor proportion of the total cost of care. A national high-cost protection scheme further ensures that individuals are not required to pay more than a specified maximum amount for healthcare services within a given period. Healthcare services are therefore accessible to all individuals.

### Recruitment and participants

The inclusion criteria for this study were healthcare professionals permanently working at the orthopaedic wards at the two hospitals. Employees at the two orthopaedic wards received information about the study at workplace meetings or in an email sent by MHN. The 19 staff members who expressed an interest in participating in the study received an information letter and gave written informed consent. The participants were informed that a physiotherapist employed at a geriatric clinic (AH) would conduct the interviews. All 19 staff members were contacted by AH via email to schedule an interview. Two withdrew their consent and one did not reply to the email. Detailed information about the participants is shown in Table 1.

**Table 1:** Demographic characteristics of the participants

Variables	(*N* = 16)
Age, mean *(min-max)*	41 (24–63)
Sex, n (%)	
Female	14 (88)
Male	2 (12)
Profession, n (%)	
Nurse	8 (50)
Assistant nurse	3 (19)
Physiotherapist	1 (6)
Occupational therapist	1 (6)
Physician	1 (6)
Counsellor	2 (13)
Specialist education, yes/no, n (%)	3/13 (19/81)
Manager yes/no, n (%)	2/14 (12/88)
Experience in profession, years, mean (min-max)	13.6 (3–36)
Experience in orthopaedics, years, mean (min-max)	8.3 (1.5–27)

### Data collection

Data were collected via face-to-face interviews using a semi-structured interview guide. The interview guide was designed by all authors and was based on open-ended questions unrelated to specific orthopaedic diagnoses, to encourage the participants to talk freely. To ensure that the interview guide was relevant, to train the interviewer, and to increase the credibility of the study, two pilot interviews were conducted with two healthcare professionals at a geriatric clinic by AH. After the first pilot, some questions were rephrased for improved comprehensibility, and an additional item regarding frailty at home or in hospital was included, to generate more in-depth responses. The pilot interviews are not included in the results. Examples of questions are: *Can you tell me how you would describe a person who is frail? What does it mean to you when you receive a report that a patient is frail?* Follow-up questions were asked to get participants to clarify or expand on their answers.

A total of 16 individual face-to-face interviews with participants (14 women; 2 men) were conducted by AH during February to May 2025. The interviews were held in secluded spots near the participants’ workplace during their shifts; each interview lasted between 13 and 34 min (median 21, IQR 16–26). The interviews were digitally audio recorded using a Sony ICD-PX470 voice recorder. Immediately after the interviews, the data were transferred to a protected data folder with a coded name and deleted from the audio recorder. The interviews were transcribed verbatim by a research administrator. One of the study participants made a short addition after the interview by email to AH.

### Reflexivity

All authors are females. AH and YN are registered physiotherapists, and MHN and EF are registered nurses. AH has long clinical experience working with geriatric patients. EF and MHN have extensive clinical experience in orthopaedic care and YN in the field of neurology. YN, MHN and EF are experienced researchers in qualitative methods. AH and YN had no ongoing association with the participants. EF and MHN were or had been employed at the orthopaedic department but were not involved in the data collection.

### Analysis

The analysis was performed considering the six phases of the thematic analysis [23]. To become familiar with the data, AH compared the transcripts with the recordings to verify their accuracy. Then AH and MHN read all the transcripts and YN and EF read eight each. Short notes were taken and a naïve understanding, inspired by the phenomenological–hermeneutic tradition [24], for each interview and later all interviews together was written down and discussed between the authors. AH, MHN, and YN did a reflexivity exercise to increase self-awareness inspired by Brown and Clarke [25]. Initially, each author reflected on their social privileges and positionalities, and how these had shaped their worldview. They then reflected upon how their research background and professional training might have influenced the interpretation of the data set. AH first conducted a manifest-level coding of the material, followed by a latent-level coding. The data were processed using Excel. To increase trustworthiness of the study, AH and MHN read and separately coded one interview at the beginning of the analysis process. The results were discussed to facilitate a more in-depth analysis of the coding process. AH proceeded to code the remaining interviews and continuously discussed the process with MHN. The next step was to search for preliminary subthemes and combine them into preliminary themes, a process in which all authors participated. This phase of the analysis ended up with a preliminary thematic map. In the next phase the preliminary themes and subthemes were critically reviewed by all authors and refined moving back and forth between the whole data set, meaning units, codes, subthemes and themes, with the naïve understanding as a backdrop. During this phase the final themes and subthemes were developed. The processes of searching for themes, reviewing and defining, involved the active participation of all four authors to ensure analytical rigour.

## Results

**Fig. 1:**
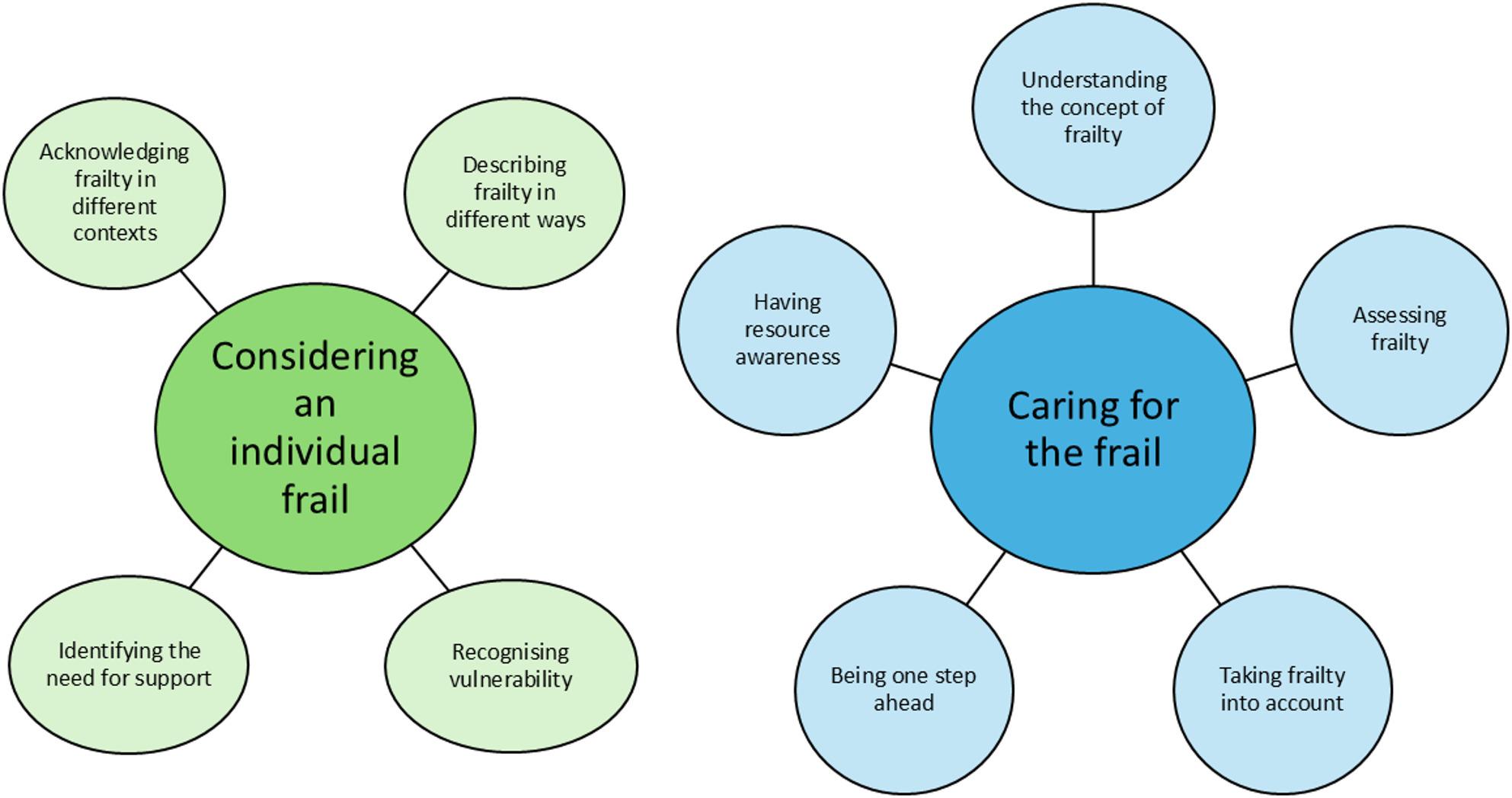
Final thematic map illustrating themes and subthemes exploring healthcare professionals’ perspectives on frailty

Two themes were developed during the analysis: “*Considering an individual frail” and “Caring for the frail”*; these were supported by nine subthemes. These themes and subthemes are illustrated in Fig. 1.

### Considering an individual frail

This theme explores healthcare professionals’ perceptions of frail individuals and the different forms of vulnerability they may experience in different contexts.

#### Describing frailty in different ways

The participants described various forms of frailty and indicated that an individual may experience multiple forms of frailty simultaneously. They explained that frailty may manifest in physical forms, such as multimorbidity, reduced mobility, increased risk of falls, or fragile skin. It may also manifest in psychological forms, including mental illness, anxiety, or the effects of previous trauma. Healthcare staff pointed out that frailty may also have a social dimension, such as loneliness, a weak social network, or financial vulnerability. Finally, a cognitive dimension of frailty was also described, which may be characterised by impaired memory and a reduced ability to understand and process information. Several participants described frailty as being present at all ages but noted that it may take different forms depending on an individual’s age.

*Well*,* but say that it’s someone my own age*,* in their forties. Then I think more that it’s about emotional fragility. Whereas if it had been a ninety-year-old I think more that it’s physical fragility*,* it might be the skin that is really fragile*,* that it breaks as soon as you touch it*,* or that [the person] is malnourished and there’s dizziness*,* like it’s kind of two different people who appear in front of me when I think about it.* – Participant 11.

#### Recognising vulnerability

Participants also identified additional characteristics of frailty. Frailty was described as having limited awareness of one’s situation and not always knowing what is best for oneself. They further described frail individuals as having diminished resilience, limited reserves and a heightened sensitivity to change, where even minor events could become overwhelming. Some participants noted that frail individuals may become caught in a frailty spiral, in which their existing condition of vulnerability leads to increased exposure to further risks and potential harm. Another indication of vulnerability was described as the need for support in performing daily activities, accompanied by challenges in maintaining sufficient nutritional intake.

*Everything from if the oral status isn’t good and then you can’t eat*,* and if you can’t eat then maybe you get tired and weak and then maybe you get falls*,* like it all kind of goes into each other. Then maybe you lie down a lot and then you get some pressure ulcers. So*,* all of this kind of goes into each other in some way.* – Participant 14.

#### Identifying the need for support

Healthcare professionals described how frail individuals may experience difficulties in initiating the necessary contacts to obtain appropriate help and support. They expressed that frail individuals can be uncertain about how to proceed or where to turn for assistance. They described how a frail individual’s request for help, when denied or delayed, could discourage future help-seeking. They also expressed that frail individuals may lack adequate support and assistance at home yet have limited ability to change their situation on their own. Some participants additionally pointed out that seeking help constitutes a considerable personal challenge. Participants also described frail individuals as more vulnerable as they may be less seen and therefore be at risk of being forgotten.

*Because it can also be a big step to ask for help. The person maybe feels that some things are starting to fail but it’s still hard to take this step to ask for help because you’re used to taking care of yourself and managing on your own*,* and so.* – Participant 3.

#### Acknowledging frailty in different contexts

The setting in which a frail individual is located—whether at home or in hospital—may entail different advantages and disadvantages. The healthcare staff described how being at home offers frail individuals several advantages, such as reduced vulnerability, increased sense of safety, a familiar environment, and higher levels of activity. But being in hospital also offers certain benefits, such as easier identification of frailty, a more holistic view of their situation, reduced loneliness, access to more care, and greater opportunities to receive appropriate support. But there were also descriptions of disadvantages to being frail, both at home and in hospital. The participants noted that in the home setting there is an increased risk of insufficient support, and frailty may be more easily concealed or remain undetected. In the hospital setting, frail individuals may become more vulnerable, receive help with activities they could otherwise manage independently, and face additional risks associated with an unfamiliar environment.

*…and this environment also makes you become more help-dependent*,* like you easily get into not getting up and not having the energy and you lose muscles and strength and you get help with things that you maybe would*,* the little you would be able to [do yourself]*,* or you get help with things you can [do yourself]*,* and then it becomes a little habit. -* Participant 17.

### Caring for the frail

This theme explores healthcare professionals’ perspectives on the concept of frailty, working with frail individuals, and frailty assessment.

#### Understanding the concept of frailty

Across the interviews, participants described frailty as a multifaceted, difficult and complex concept. They repeatedly emphasised the breadth of the term, noting that it encompasses a wide range of possible meanings. Participants also highlighted that the term does not adequately capture the specific needs or circumstances of individual patients and is open to multiple interpretations. Understanding the unique manifestation of frailty in each patient was considered crucial. Some participants also pointed out that the perception of being frail can carry negative connotations, as one does not wish to be considered frail or to be seen as a burden. One participant emphasised, however, that having limitations does not necessarily mean being frail.

…*like the word “frail” doesn’t really tell me what I need to do for this individual. But if you just give me that word then*,* uh*,* I really need to dive deep into that person to actually see what it is you mean by frail in this individual. It’s a big*,* broad concept*,* really.* – Participant 13.

#### Assessing frailty

The participants indicated that the frailty assessments with the CFS concerned individuals aged 65 years and older and were based on their level of functioning 14 days prior to hospital admission. The assessments were conducted at admission or during ward rounds, although some participants expressed that it could also have been performed by the ambulance service or in the emergency department. Several participants pointed out that some of the frailty assessments performed needed to be adjusted during subsequent ward rounds. Different views were expressed regarding which professional groups were involved in assessing frailty; in some cases it was done by those in a specific profession, and in others it involved the entire team. To assess frailty, information about the patient’s previous functional level was obtained from the patient, relatives or the municipality staff. Several participants expressed that they would have preferred to meet the patient before performing the assessment.

The healthcare staff described some challenges with frailty assessment. Some found the CFS difficult to use and that it was helpful when several colleagues carried out the assessment together. They also indicated that whether the assessment was performed, and if so, how easy or difficult it was to do so depended greatly on which physician attended the ward round. There was considerable variation in how well the physicians were onboarded to the frailty assessment; the more familiar they were with the process, the easier it became, and vice versa. Many participants reported that it was easy to overlook and deprioritise the frailty assessment at admission, as other tasks were given higher priority. The participants expressed several advantages of assessing frailty. They perceived the CFS as an important tool for identifying frailty and clarifying the needs of frail individuals. They described how frailty assessment could prompt a broader discussion of frailty, enabling the collaborative formulation of plans that identify priority interventions. Some also regarded the assessment as helping to prevent hospital readmissions and as an opportunity to develop and follow concrete care plans based on the level of frailty. It also made it possible to allocate resources more appropriately and to avoid overtreatment. The participants also revealed reflections indicating that staff had started to think one step further about frailty and had gained an increased understanding of frail patients since the introduction of frailty assessments. It was regarded as a means to improving the quality of care. One participant noted that the level of frailty can be more informative than chronological age.

*At first*,* I thought that this kind of feels a bit cumbersome and just one more thing you have to do. But when you*,* like*,* dive deep into this*,* then it’s really about driving healthcare forward in some way.* – Participant 13.

*I think it’s good in a way with the CFS*,* that it’s becoming more and more integrated*,* even if unfortunately sometimes… well*,* many people might forget it*,* during admission*,* so it gets a bit overlooked. But I do think we’re good at reminding each other; in each ward round room we’ve put up a little note saying that patients should be CFS-assessed. Still*,* I believe it’s good that we are working more with it*,* so that we can identify individuals who are frail. –* Participant 8.

#### Taking frailty into account

The staff expressed that they felt a greater sense of responsibility for frail patients. They emphasised their role in representing and advocating for the needs of frail individuals, who may have limited ability to express these needs on their own. The staff described a wish to provide frail individuals with additional care and emotional support. Although a frailty assessment had been carried out for the patients, the participants emphasized that their approach was mostly adapted to the needs of the individual and not specifically to the level of frailty. The participants described how they at times adapted and modified their way of working due to patients’ frailty, however. This could involve allocating more time to frail patients, who often require increased nursing. They also provide extra support, helping the patients conserve their energy for essential tasks, and they were careful to communicate more clearly and explicitly. Some participants also expressed that frail individuals are particularly vulnerable to rapid care processes, ward relocations and alterations in surgical schedules. One participant expressed a desire for patients to be prioritised based on their frailty. They saw it as crucial to take a holistic approach rather than focusing exclusively on medical aspects. Participants emphasised the importance of providing frail individuals with adequate support and resources to preserve their abilities and restore their functional level. The staff explained that discharge planning is commonly carried out for frail patients, often involving family members. It was occasionally found necessary to adjust the approach during discharge planning to guarantee that the frail individuals would receive adequate care and support, as well as access to assistive devices, after leaving the hospital.

*It feels like we have an important role in helping those patients. Because many times it’s not the frail patients who take up the most space.* – Participant 5.

#### Being one step ahead

The healthcare staff described the need to be one step ahead in order to provide high-quality care for frail individuals. Risk prevention and preventive measures were seen as essential parts of caring for frail individuals. These include preventing malnutrition by monitoring dietary and fluid intake and providing nutritional supplements or parenteral nutrition when needed. Maintaining a preventive mindset was regarded as important, particularly in relation to pressure ulcer prevention, mobilisation, and the management of confusion. Frail individuals may require further optimisation, both pre- and postoperative. The staff expressed an increased responsibility for evaluating patients’ needs and well-being. Accurate reporting of a frail patient’s condition was described as crucial, and the staff felt a strong responsibility to provide comprehensive and detailed handovers, as the patient might not always be able to advocate for themselves. Some participants explained that the term “frail” was not necessarily used in handovers but that the implications of frailty were still communicated. Some also expressed the importance of reviewing medication lists and ensuring safe medication management before discharge. Participants expressed ideas about preventing and addressing frailty in the home setting to reduce the risk of hospital admission.

…*like check the patient carefully. And*,* to put in preventive measures and try to help so that nothing bad happens or [being aware] that there is a higher risk that*,* as said*,* something could happen with the patient. And keep an extra watch and work more preventively*,* I would do [that].* – Participant 5.

#### Having resource awareness

The participants pointed out that caring for frail patients requires increased staffing levels since these patients often need closer supervision and more extensive care interventions. Caring for frail patients often requires a multidisciplinary team involving several professional categories, allocating more resources to those with the greatest needs. Many participants regarded teamwork as essential when caring for frail patients. The care of frail patients demanded more frequent communication between staff members and closer collaboration concerning mobility and assistive devices. According to the participants, discharge planning for a frail individual necessitates the involvement of a wider group of professionals.

*Many times*,* the frailer the patients are*,* the more help they need to have*,* the more staff we need to get them to function*,* kind of.* - Participant 16.

## Discussion

This study provides insight into how healthcare professionals at an orthopaedic ward understand the concept of frailty, perceive frail patients and adjust their care practices in response to patients’ frailty. Our analysis shows that participants perceived frailty as a complex, multidimensional construct which necessitates adaptation of healthcare. Participants’ perceptions of frailty do not fully align with the WHO’s definition, as they, for example, emphasise social aspects of frailty such as loneliness, a weak social network or financial vulnerability [3]. It’s an interesting finding; it shows that the concept is interpreted broadly and extends beyond the WHO’s definition. An awareness that this concept holds different meanings for different individuals highlights the need to remain attentive to how the term frailty is used in communication between colleagues and when introducing newly employed staff. The concept seems to require a clearer specification to guide appropriate understanding and interventions. Our findings also indicated that staff did not consistently take the individual’s level of frailty into account when adapting their interventions; instead, they tended to base their actions on the individual’s immediate needs. Working in a person-centred manner with older adults has been shown in previous research to be an effective approach [26, 27]. It is not suitable to standardise all aspects of care, as needs are highly individual, varying considerably not only between individuals but also for the same individual at different times as their care needs change throughout the day, and it is essential to support patients in maintaining the greatest possible level of independence. Prior research indicates that adopting a person-centred approach in the care of older adults enhances staff satisfaction and strengthens their perceived ability to address individual needs [28]. Nevertheless, the ability to provide person-centred care is dependent upon staff having access to suitable conditions, including supportive environments, adequate staffing, and balanced workloads [16].

Furthermore, it became apparent that the participants felt a strong sense of responsibility for this patient group, noting that frail individuals require substantial staffing resources to avoid being overlooked or exposed to additional risks and harm. This is an important consideration for future staffing strategies in units that care for a high proportion of frail patients [29] and to reduce moral distress [30].

Participants’ statements indicating that many frail individuals who are admitted to the hospital receive insufficient support at home and arrive from unsustainable home situations are concerning. The participants perceived that many frail individuals either do not know how to seek help or are unable to do so. Admission to hospital therefore presents an opportunity for them to receive necessary support and assistance at home, which makes care planning at discharge for frail individuals fundamental. Research indicates that older individuals desire services that are more responsive to their personal needs, together with stronger coordination of care, such as during hospital discharge [31]. Considerable responsibility is placed on healthcare professionals, society, and family members to intervene early and provide support before the situation deteriorates. Participants described not always having the time to assume this responsibility due to the fast pace of care work which relates to the findings of a previous study indicating that high patient turnover can impair staff’s ability to provide safe and high-quality care [32].

Several components of frailty described by the healthcare professionals are not fully captured by the CFS, which indicates that the CFS cannot, on its own, serve as a sufficient instrument for managing frailty in an orthopaedic setting. Neither simply identifying an individual as frail nor the CFS score itself provides insight into an individual’s specific care or support needs, but the assessment nevertheless appeared to function as a useful prompt for deeper conversations about frailty. This aligns with findings from a prior study that revealed that the CFS is perceived as a shared tool for communication and collaborative work within the care team [15]. Participants valued this aspect, noting that the CFS encouraged more systematic reflection on frailty and on the needs of individuals living with frailty. This is important, as other studies indicate that inpatient care settings risk focusing solely on the aspect of frailty that pertains to their own discipline [21]. Other studies suggest that implementation of the CFS and Comprehensive Geriatric Assessment (CGA), a treatment approach for the structured management of frail older adults, would likely further improve care and reinforce the approach to managing frailty [15, 27].

### Strengths and limitations

We followed Brown and Clarke’s six phases for thematic analysis [23] but also employed naïve understanding inspired from the phenomenological-hermeneutic tradition [24]. Reflexivity was a crucial and continuous component of the analysis process, where all authors participated. All authors were involved in the analysis to enhance the trustworthiness of the analyses and the final themes.

A potential limitation is that two of the co-authors, MHN and EF, work or have previously worked at the orthopaedic clinic, which could have influenced participants’ willingness to respond candidly. While this does not appear to be the case, as several participants raised concerns and were very open about their practices, the risk cannot be fully discounted. To enhance the credibility of the findings, it was decided that the person currently working at the orthopaedic clinic would not handle recruitment or conduct the interviews. On the other hand, interest in participating in the study may increase when individuals involved in the project have a thorough understanding of the organisation [33]. Another potential limitation was that AH was a novice interviewer. This was mitigated through two pilot interviews, during which the authors jointly reviewed the recordings to ensure consistency and quality. Given that the resulting interview data demonstrated substantial richness and depth, this factor is not considered a limitation.

There is a possibility that participants with alternative viewpoints were not included, either due to the staff’s constrained schedules, concerns about expressing opinions that differed from the norm, or the belief that they had nothing valuable to contribute, even though they did. However, there was considerable variation in terms of professions, age, and years of experience both in the profession and at the workplace, indicating that a large portion of the workplace was still represented. The relatively modest sample size may also be viewed as a constraint. Nevertheless, there was variation in the demographic factors among the participants, while the responses showed a high degree of consistency, described as information power [34]. This study included participants from two hospitals within the same region of the same country, which may limit the transferability of the findings.

## Conclusions

This study highlights that healthcare professionals view frailty as a broad and multidimensional concept that requires clearer specification to guide appropriate interventions. The findings underscore that care is primarily adapted to the individual rather than guided by a frailty assessment, reflecting a person-centred approach. Professionals also expressed a strong sense of responsibility toward frail patients and indicated that hospital admission could present frail individuals with an opportunity to receive necessary assistance at home. Finally, while the CFS were perceived not to capture all aspects of frailty, it was described as a useful instrument for directing staff attention to frailty and facilitating more in-depth discussions about the concept.

## Supplementary Information

Supplementary Material 1.

Supplementary Material 2.

## Data Availability

The dataset generated during the current study is not publicly available due to the protection of individual privacy but is available from the corresponding author on reasonable request.

## Abbreviations

CFS: Clinical Frailty Scale
WHO: World Health Organization
CGA: Comprehensive Geriatric Assessment
IQR: Interquartile Range
