# Supplementary material for: Caring for the frail: a qualitative study in an orthopaedic setting
Source: BMC Geriatr. 2026 Apr 16;26:548. doi: 10.1186/s12877-026-07479-7 (PMC13088482; doi:10.1186/s12877-026-07479-7)
Supplement: Supplementary file 2 — Supplementary Material 2. [file 12877_2026_7479_MOESM2_ESM.docx]

**Interview guide**

- Age?
- Education? Specialist education?
- Years of experience in profession?
- Years of experience in orthopaedics?
- Manager?
- Any other relevant competencies?
- Can you describe how you would portray a person who is frail? It does not have to be a patient, just a frail person in general.
- If I were to say that a person is frail – what thoughts would you have about that person?
- All individuals who come to the orthopaedic department are assessed regarding their level of frailty, and the care is adapted based on that assessment. How have you been involved in this?
- What are your thoughts on tailoring care to a person’s degree of frailty?
- What does it mean for you in your work to be informed that a person is frail — in collaboration with colleagues, in patient interactions, during discharge, etc.? How does it differ compared to someone who is not frail?
- Do you think there may be differences between someone who is frail and living at home versus someone who is frail and admitted to the hospital? Tell me more.
- Is there anything else you would like to add or talk about?
